# Supplementary material for: Prerequisite Binding Modes Determine the Dynamics of Action of Covalent Agonists of Ion Channel TRPA1
Source: Pharmaceuticals (Basel). 2021 Sep 28;14(10):988. doi: 10.3390/ph14100988 (PMC8540651; doi:10.3390/ph14100988)
Supplement: Supplementary file 1 [file pharmaceuticals-14-00988-s001.zip › pharmaceuticals-1371563-supplementary.pdf]

Supporting Information for

Prerequisite binding modes determine the dynamics of action of covalent agonists of ion channel TRPA1

Balázs Zoltán Zsidó[1], Rita Börzsei[2], Erika Pintér[1], Csaba Hetényi[1*]

[1] Department of Pharmacology and Pharmacotherapy, Medical School, University of Pécs, Szigeti út 12, 7624 Pécs, Hungary.

[2] Department of Pharmacology, Faculty of Pharmacy, University of Pécs, Szigeti út 12, 7624 Pécs, Hungary

* Correspondence: hetenyi.csaba@pte.hu

Table S1 Covalent docking calculations performed by FITTED on the apo target with A-loop

Ligand	JT010	BITC	bodipy-iodoacetamide
Rank	1/10	1/10	1/10
ΔG_{FD} (kcal/mol)	**+88.27!**	-66.2	-9.91
NHA[c]	23	10	22
EI_{NHA}[d]	3.84	6.62	0.45
RMSD of Rank 1 (Å)	10.00	9.29	11.75
$d_{covalent}$ (Å)	1.8	1.8	1.8

Table S2 Non-covalent docking calculations performed by FITTED on the apo target with A-loop

Ligand	JT010	BITC	bodipy-iodoacetamide
ΔG_{FD} (kcal/mol)	**+969.58!**	-15.15	**+405.97!**
$Rank_{best}$	1/10	1/10	1/10
d_{best} (Å)	3.3	3.5	4.3

Table S3 Non-covalent docking calculations performed by AutoDock on the apo target with A-loop

Ligand	JT010	BITC	bodipy-iodoacetamide
ΔG_{AD} (kcal/mol)	-4.82	-4.79	-5.08
$Rank_{best}$	1/5	1/3	1/3
d_{best} (Å)	10.0	11.4	9.8

Table S4 Interacting (≤3.5Å) amino acid residues of 6PQO with the non-covalent top ranked binding mode of JT010 (FITTED)

Interacting amino acid residues (6PQO)	JT010 non-covalent	JT010 crystallographic
L609	X	X
K610	X	X
F612	X	X
H614		X
C621	X	X
P622		X
I623	X	X
T624		X
K661	X	X
Y662	X	X
Q664	X	X
C665	X	X
P666	X	X
Y680	X	
T684	X	
A685		

Table S5 The interacting amino acids (within 3.5 Å) of the experimental binding position, the covalently docked and prerequisite binding modes of BODIPY-IODOACETAMIDE.

	Covalent docking		Prerequisite docking			
bodipy-iodoacetamide experimental	bodipy-iodoacetamide holo FD	bodipy-iodoacetamide apo FD	bodipy-iodoacetamide holo FD	bodipy-iodoacetamide apo FD	bodipy-iodoacetamide holo AD	bodipy-iodoacetamide apo AD
C621	L609	F612	L609	L609	L609	N615
C665	F612	H614	K610	K610	H614	S616
P666	H614	N619	F612	F612	I623	K620
	K620	K620	H614	H614	K661	T624
	C621	C621	C621	C621	Y662	Y680
	P622	P622	I623	I623	Q664	E681
	I623	I623	K661	T624	C665	
	T624	T624	Y662	Y662	P666	
	E625	E625	L663	Q664	F669	
	K661	C665	Q664	T684	T684	
	Y662	V678	C665			
	Q664	Y680	P666			
	C665	E681	F669			
	P666	P682	Y680			
	F669	L683	T684			
	Y680	T684				
	T684					
AAmatch	100%	66.60%	100%	33.30%	66%	0%

Table S6 The interacting amino acids (within 3.5 Å) of the experimental binding position, the covalently docked and prerequisite binding modes of BITC.

	Covalent docking		Prerequisite docking			
BITC experimental	BITC holo FD	BITC apo FD	BITC holo FD	BITC apo FD	BITC holo AD	BITC apo AD
C621	K620	F612	L609	F612	L609	S613
I623	C621	H614	C621	H614	C621	H614
K661	P622	N619	I623	C621	I623	N615
Y662	I623	K620	T624	I623	T624	S616
Q664	T624	C621	K661	T624	Y662	K620
	K661	P622	Y662	Q664	Q664	I679
	Y662	I623	Q664	C665	T684	Y680
	Q664	T624	C665	Y680	A685	
	C665	E625	T684	T684		
	T684	Q664				
		C665				
		Y680				
		T684				
AAmatch	100%	60%	100%	60%	80%	0%

Table S7 The interacting amino acids (within 3.5 Å) of the experimental binding position, the covalently docked and prerequisite binding modes of JT010.

	Covalent docking		Prerequisite docking			
JT010 experimental	JT010 holo FD	JT010 apo FD	JT010 holo FD	JT010 apo FD	JT010 holo AD	JT010 apo AD
C621	L609	L609	L609	L609	C621	H614
T684	K610	F612	K610	K610	I623	N615
	F612	N619	F612	F612	T624	S616
	H614	K620	H614	H614	K661	K620
	K620	C621	C621	C621	Y662	T624
	C621	P622	P622	I623	Q664	I679
	P622	I623	T624	T624	C665	Y680
	I623	T624	K661	K661	Y680	E681
	T624	E625	Y662	Y662	T684	T684
	E625	K661	Q664	Q664		N687
	K661	Y662	C665	V678		A688
	Y662	Q664	P666	Y680		
	Q664	C665	F669	T684		
	C665	Y680	Y680			
	P666	E681	T684			
	T684	P682				
		L683				
		T684				
AAmatch	100%	100%	100%	100%	100%	50%

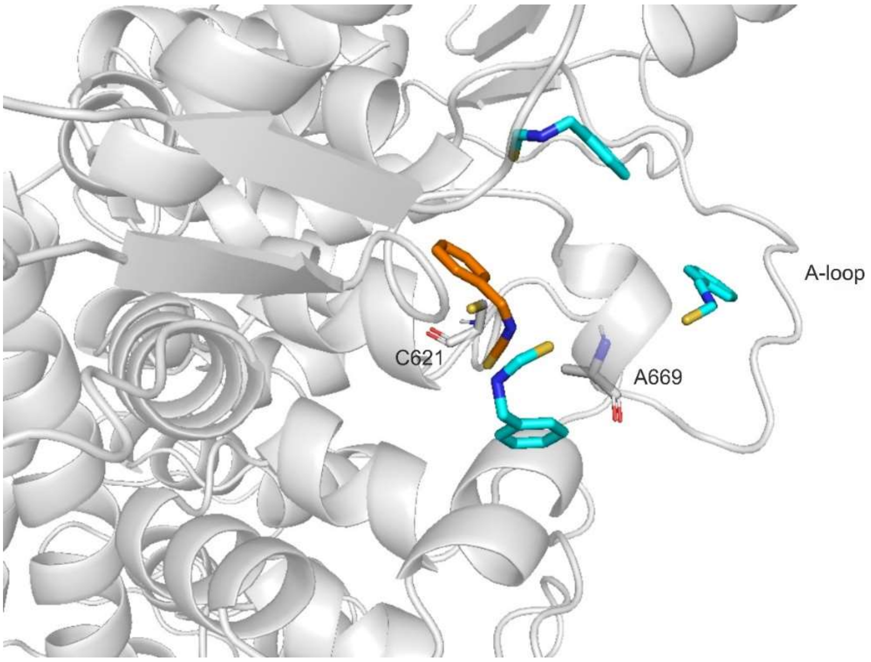

Figure S1 The close-up of binding of BITC to TRPA1 mutant structure. The docked ligand binding modes of BITC to the wild type TRPA1 receptor are shown as teal sticks for comparison, and the docked ligand binding mode of BITC to F669A TRPA1 is shown is orange sticks. Without the blocking effect of F669 in the outer prerequisite binding mode, the inner binding pocket shows increased accessibility. The mutated A669 and the wild type C621 are shown as grey sticks, the TRPA1 protein is shown as grey cartoon.
